# Risk-Reducing Salpingo-Oophorectomy (RRSO) Combined with Simultaneous Mastectomy in Women with BRCA 1–2 Mutation Carriers: The Surgical Technique, the Feasibility and Patients’ Satisfaction of Multiple Surgeries

**DOI:** 10.3390/jcm11247502

**Published:** 2022-12-18

**Authors:** Carlo Saccardi, Giulia Spagnol, Tania Saibene, Luciana Serena De Lorenzo, Matteo Marchetti, Giulio Bonaldo, Silvia Michieletto, Maria Cristina Toffanin, Marco Noventa, Roberto Tozzi

**Affiliations:** 1 Department of Women and Children’s Health, Clinic of Gynecology and Obstetrics, University of Padua, 35100 Padua, Italy; 2 Breast Surgery Unit, Veneto Institute of Oncology IOV—IRCCS, 35100 Padua, Italy

**Keywords:** risk-reducing salpingo-oophorectomy (RRSO), risk-reducing bilateral mastectomy (RRBM), BRCA 1–2, combined surgeries

## Abstract

The main goal of our study was to evaluate the surgical technique, the feasibility and patient’s satisfaction of multiple surgeries: Risk-reducing salpingo-oophorectomy (RRSO) combined with mastectomy in patients with BRCA 1–2 mutation carriers. We conducted a retrospective analysis of patients with BRCA 1–2 variants who underwent RRSO combined with risk-reducing bilateral mastectomy (RRBM) or surgeries for breast cancer from January-2015 to December-2021. We collected data about surgeries, complications, and patients’ satisfaction using a questionnaire submitted 30 days after surgery. We included 54 patients. Forty-eight patients underwent RRSO, and six patients underwent RRSO + Total laparoscopic hysterectomy (LTH). The minor postoperative complications within 30 days were four: one breast seromas aspiration (1.9%), one infectious reconstructive complication treated with antibiotics therapy (1.9%), one Red-Breast-Syndrome (1.9%) and one trocar abdominal hematoma (1.9%) associated with RRSO. The major postoperative complications within 30 days were five: two evacuations of a breast hematoma (3.7%) and three infectious reconstructive complications treated with removal expander/implant (5.6%). No postoperative complications after 30 days were observed. According to the satisfaction questionnaire, more than 90% of patients were satisfied and would have combined surgery again. In conclusion, the multiple surgeries seem feasible and safety with a single anesthesia, a single surgical time, a single postoperative recovery, and a high patients’ satisfactions without increasing morbidity.

## 1. Introduction

Germline mutations in the BRCA1 and BRCA2 genes correlate in the development of most cases of hereditary breast and ovarian cancer [1]. In particular, the lifetime risk of breast cancer (BC), for BRCA 1–2 mutations carriers is 40–85% by the age 70 [1,2,3,4]; whereas the risk of ovarian cancer (OC) (including fallopian tube cancer and primary peritoneal cancer) for BRCA1 mutation is 39–46% by the age 70 and for a BRCA2 mutation is 10–27% by the age 70 [1,5,6]. The cumulative risk of BC in BRCA1 and BRCA2 mutation carriers is 72% and 69%, respectively, by the age of 80, and for OC is 44% and 17%, respectively, by the age of 80 [7].

According to the National Comprehensive Cancer Network (NCCN), the risk-reducing salpingo-oophorectomy (RRSO) and the risk-reducing bilateral mastectomy (RRBM) can decrease the lifetime risk of developing OC by more than 95% and BC by 85–90% in patients with BRCA1–2 mutation carriers [8]. RRSO is recommended at age 35–40 years for BRCA1 carriers, whereas women with BRCA2 carriers may consider delaying until age 40–45 years [9,10,11,12]. Some authors affirmed that breast therapeutic surgeries or RRBM combined with RRSO can easily be performed in patients with BRCA mutation carriers [13]. However, many patients are uncertain about undergoing combined surgeries. In fact, in literature there are limited data about the feasibility and safety of combined surgery and no studies evaluate patient’s satisfaction [14,15,16]. Therefore, the aim of our study was to evaluate the surgical technique, the feasibility and patient’s satisfaction after RRBM or breast therapeutic surgeries for BC combined with simultaneous RRSO in patients with BRCA 1–2 mutation carriers.

## 2. Materials and Methods

### 2.1. Study Design and Inclusion/Exclusion Criteria

We conducted an observational retrospective cohort study on patients with documented BRCA1–2 germline variants who had undergone RRSO combined with RRBM or therapeutic surgeries for a new or previously diagnosis of BC at Clinic of Gynecology and Obstetrics of University of Padova-Department of Women and Children’s Health and Breast Cancer Unit of Veneto Institute of Oncology IOV–IRCCS.

The surgeries were performed between January 2015 to December 2021. All women enrolled in this study had a proven germline BRCA1–2 variants. All patients signed a document approved by our institution for the anonymous use of their clinical data for scientific purposes according to the European privacy law. The institutional review board approved the study (87n/AO/21).

The inclusion criteria were as follows: patients with BRCA1–2 variants diagnosed through genetic testing performed by blood sampling; patients that underwent RRSO combined with breast surgery in the setting of a new diagnosis of BC and/or in the setting of RRBM combined, according to NCCN guidelines [8]; asymptomatic women with a negative pelvic examination and transvaginal ultrasound at the last screening prior to RRSO (3–6 months before surgery). Exclusion criteria: patients with a positive gynaecologic screening (adnexal mass at transvaginal ultrasound or positive CA 125) or women with ovarian/tubal cancer prior to RRSO, no confirmed pathogenic BRCA1–2 variants.

### 2.2. Data Collection and Statistical Analysis

Patients were identified through our institution computer database initiated to collect clinical and surgical information at the point of care. For each patient the investigators reviewed the electronic hospital records and pathology reports to determine study eligibility, patients’ general features and histopathological features. In cases of missing information, investigators directly contacted the patient by telephone or email to complete the data collection.

We collected data on age, menopausal status at RRSO, family history of BC and OC, surgical technique of mastectomy (uni- or bilateral; therapeutic or prophylactic) and reconstructive details (one stage/two stages reconstruction), time of breast surgeries and RRSO and duration of hospitalization. We also collected data about surgical perioperative complications subdivided into intraoperative complications, early postoperative complications (in the first 30 days) and late postoperative complications (after 30 days—i.e., postoperative infection, hematoma, flap necrosis, and failed reconstruction, urinary retention, abdominal wall abscess). We divided the complications into a major when an added surgeries occurred (i.e., implant failure or breast reconstruction/revision) and a minor when a patient required antibiotics, local therapy, or blood transfusion.

During the first follow-up visits at 30 days, we submitted to all patients a questionnaire to evaluate general subjective satisfaction with the combined surgical approach.

### 2.3. Endpoints of the Study and Statistical Analysis

The primary endpoint of this study was to evaluate the surgical technique, the safety and feasibility of combined surgeries: RRSO with simultaneous (i) Bilateral mastectomy: RRBM or therapeutic surgery with or without immediate reconstruction or (ii) Unilateral mastectomy: prophylactic or therapeutic surgery with or without immediate reconstruction. Other endpoints were the following: evaluate patients’ satisfaction with the combined surgical approach, postoperative pain and recovery time.

The statistical analysis was performed with the SPSS Software (Chicago, IL, USA) for Windows version 26. Continuous variables (distributed normally) were expressed in absolute numbers and mean ± standard deviation, Categorical variables were expressed as absolute numbers and percentages.

### 2.4. Surgical Technique

The surgery was subdivided into two surgical times: the first performed by a gynaecologist and the second by a general surgeon and a plastic surgeon, during the same anaesthesiologic session. In particular:

(I) RRSO is a minimally invasive surgical procedure performed laparoscopically following NCCN guidelines [8]. The patient was placed in the Trendelenburg position. The laparoscopic camera was inserted through the umbilical trocar and all other trocars were inserted under direct vision. The diaphragm, liver, omentum, bowel, paracolic gutters and appendix were inspected in the abdomen. The ovaries, fallopian tubes, uterus, bladder serosa, and cul the sac were inspected in the pelvis. The first part consisted of peritoneal washing (PW) collected for cytologic examination. Then we performed a laparoscopic salpingo-ophorectomy as follows: we opened the posterior broad ligament to access the retroperitoneum and to view the ureter course; we coagulated and cut through utero-ovarian ligament and proximal fallopian tube at the level of the uterine wall, dissecting the mesosalpinx and separating the adnexa from the pelvic wall; we coagulated and cut the infundibulopelvic ligament. The ovarian vessel was isolated and ligated approximately 2 cm proximal to the end of identifiable ovarian tissue to ensure that all ovarian and tubal tissue is completed removed. Both ovaries, fallopian tubes, and mesosalpinx were removed inserting an endocatch bag and retrieving the adnexa. After counselling, concomitant laparoscopic total hysterectomy (LTH) was offered to the patients in case of family history of endometrial cancer and symptomatic benign uterine disease (fibroid, adenomyosis).

(II) RRBM or therapeutic surgery were performed as follows: we used different types of mastectomies, including skin-sparing mastectomy (SSM) and nipple-areolar sparing mastectomy (NSM) with or without immediate breast reconstruction. In SSM most of the breast skin was conserved to create a pocket that facilitates immediate breast reconstruction with implant to achieve a quality cosmetic outcome. Instead, in NSM the nipple-areola complex (NAC) was conserved. For an SSM, the incision may be a small ellipse around the NAC or a circular incision with or without a lateral extension if needed. NSM can be performed via a variety of incisions (inframammary, midlateral, circumareolar, or a combination) depending on breast shaping. When necessary, sentinel node biopsy was performed through a separate axillary incision. Implant-based (i.e., prosthetic: tissue expanders, silicone implants or polyurethane implants) reconstruction was performed in one or two stages: (i) one-stage reconstruction where a permanent implant was inserted at the time of mastectomy; (ii) two-stage reconstruction where a tissue expander was placed following the mastectomy and a permanent implant was used to replace the tissue expander at a later date. Prosthetic devices were placed under (subpectoral) or above (prepectoral) the pectoralis major muscle.

### 2.5. Questionnaire

We formulated a simple questionnaire based on 5-point Likert scale for question 1 and 2 and based on a binomial answer (yes/no) for the question 3, 4 and 5.

Question 1: How satisfied was she with the combined surgery? (1, Very unsatisfied; 2, Unsatisfied; 3, Neutral; 4, Satisfied; 5, Very satisfied).

Question 2: Do you believe that the postoperative pain was increased by the combined surgery? (yes/no).

Question 3: How much was the overall impact of post-operative gynecological pain? (1, Not very much; 2 Not much; 3, Neutral; 4, Much; 5, Very much).

Question 4: Do you believe that the post-operative recovery period was prolonged by having performed the combined surgery? (yes/no).

Question 5: Would you undergo combined surgery again? (yes/no).

## 3. Results

### 3.1. General Features

A total of 54 BRCA1–2 variants women underwent combined surgery from January 2015 to December 2021 at our institution. Specifically, 32 patients (59.3%) carried the BRCA1 variant, and 22 patients (40.7%) carried the BRCA2 variant. Table 1 describes the patients’ baseline characteristics. The mean age was 45 (35–72) for patients with the BRCA1 variant and 47 (42–63) for patients with the BRCA2 variant. All preoperative CA-125 level was negative. Twenty-eight (51.9%) patients presented BC at the time of surgery (current and/or recurrent). A family history of OC was observed in 18 (56.3 %) patients with the BRCA1 variant and in 9 (40.9%) patients with the BRCA2 variant; a family history of BC was observed in 19 (59.4%) patients with the BRCA1 variant and in 18 (81.8%) patients with the BRCA2 variant; a total of 3 (9.4%) women with the BRCA1 variant and 4 (18.2%) women with the BRCA2 variant had a negative family history of cancer (see Table 1 for details).

### 3.2. Surgical Technique

We performed bilateral mastectomy in 47 (87%) patients. Specifically, 22 (40%) patients underwent RRBM, and 25 (47%) patients underwent therapeutic surgery for BC. The majority of patients (*n* = 41; 76%) underwent immediate breast reconstruction with implant-based reconstruction (see Table 2 for details).

Instead, we performed unilateral mastectomy in 7 (13%) patients. In particular, 4 (7.4%) patients underwent prophylactic unilateral mastectomy, and 3 (5.6%) patients underwent therapeutic surgery for BC. Six (11%) patients underwent immediate breast reconstruction with implant-based reconstruction. Regarding gynecological surgery 48 (88.9%) patients underwent only laparoscopic RRSO and 6 (11.2%) patients underwent RRSO with concomitant laparoscopic total hysterectomy (LTH) (see Table 2 for details).

The median duration of the entire procedure (mastectomy + RRSO) was 231 ± 50 min and mean duration of RRSO was 46 (32–60) minutes. The mean duration of the entire procedure (mastectomy + RRSO + LTH) was 278 (200–345) minutes and mean duration of RRSO + LTH was 105 (90–140) minutes. The average inpatient hospital stay was 3 days (range 1–9 days). All combined surgeries were performed under a single anesthetic session (see Table 3 for details).

### 3.3. Complications

The perioperative characteristics and complications are described in Table 4.

No intraoperative complications were observed. We reported four minor early post-operative complications (<30 days that did not require surgical intervention or hospitalization): (i) breast seromas aspiration (1 patients, 1.9%) (ii) infectious reconstructive complications treated with antibiotics therapy without removal expander/implant (1 patients, 1.9%) and (iii) fever and skin infection (1 patients, 1.9%). Only one (1 patients, 1.9%) minor complication was associated with gynecological surgery represented by trocar abdominal hematoma treated conservatory without evacuation. Instead, we reported 5 early major postoperative complications that included: (i) evacuation of a breast hematoma (2 patients, 3.7%); and (ii) infectious reconstructive complications treated with removal expander/implant (3 patients, 5.6%). No major gynecological surgery complications were observed. No patients developed clinically relevant venous thromboembolism, and no perioperative mortality was observed. No late postoperative complications >30 days were observed.

### 3.4. Questionnaire

Question 1. How satisfied was she with the combined surgery? Answers: 77% of patients was very satisfied and 13% was satisfied by the combined surgery; 3% of patients was neutral and 7% was unsatisfied. No patients answered very unsatisfied.

Question 2. Do you believe that the postoperative pain was increased by the combined surgery? Answers: 95% of patients affirmed that the postoperative pain was not increased by the combined surgery.

Question 3. How much was the overall impact of post-operative gynecological pain? Answers: 77% of patients replied Not very much and 13% Not much; 8% of patients was neutral and 2% replied very much.

Question 4. Do you believe that the post-operative recovery period was prolonged by having performed the combined surgery? Answers: 88% of patients affirmed that the post-operative recovery period was not prolonged by having performed combined surgery.

Question 5. Would you undergo combined surgery again? Answers: 92% of patients affirmed yes.

(see Table 5 for details).

## 4. Discussion

In 1896, the Lancet published the first report of successful endocrine manipulation in patients with locally advanced BC who regressed after surgical oophorectomy [17]. Following that, the literature reported that RRSO can decrease BC risk by 37–100% [9,11,18], improve BC outcomes and prevent subsequent ovarian cancer in BRCA 1–2 mutations carriers with BC [18,19,20]. In light of this evidence, a significant study found that the protective effect against BC occurs only if patients are premenopausal at the time of RRSO [20]. On the other hand, one study found no reduction in BC risk associated with RRSO after using different analytics and adjusting for cancer at the time of test and time preceding RRSO [21].

Considering this important and debate issue, our study focused the attention on patients with documented BRCA1–2 mutation carriers who undergone RRSO combined with RRBM/therapeutic mastectomy to evaluate the feasibility, safety, and patients’ satisfaction of coordinating procedure.

The literature about the multispecialty surgery is poor and the published studies are potentially underpowered by the low number of patients included [14,15,16,22,23,24]. Although these few data presented an advantage in terms of reduction in separate hospitalizations, costs, and anesthetic administrations, in most centers breast surgery and RRSO are usually performed in two separate procedures due to structural problems [16,22,24].

Moreover, no studies reported data about patients’ satisfaction related to combined surgery. In fact, as underlined by previous papers, many patients are skeptical to undergo multiple surgeries that might harbor further operative risks and prefer to undergo a single procedure [22,23,24].

Our population represented one of the largest series of simultaneous breast and gynecologic surgery in BRCA 1–2 mutation carriers. Considering feasibility and safety of combined procedure, we reported a total complication rate of 18% (16.6% related to breast surgery versus 1.9% related to RRSO); however, is important to underline that almost all complications were classified as minor (treated conservatory) and only 5 patients required second surgery. The only gynecologic complication was represented by an early minor post-operative complication treated conservatory. The higher complication rate after breast surgery can be explained by the longer duration of this surgery compared to RRSO and the increased infectious risk related to reconstruction [25,26]. On the contrary, the RRSO was a minimally invasive surgery with a low rate of complications and the women was very satisfied with their choice of risk-reduction strategy [27,28,29]. In a recent large series of RRSO, authors reported only three minor complications related to anesthesia [25]. This data confirms the goodness of our combined approach, in which a single anesthetic time can reduce the total complication rate compared to the two separate surgery.

The total complication rate reported by literature ranged from 18–44% [14,15,16,22,23,24]. This wide range could be related to the small sample size of previous trials and to the different types of breast reconstruction (autologous or implant/expansor-based reconstruction).

In 2008, Batista et al. published one of the first study about 12 patients that underwent combined surgery demonstrating the safety and efficacy of a free-flap-Y based breast reconstruction with simultaneous RRSO [14]. Similarly, Willsher et al. concluded that simultaneous procedures are feasible and carry an acceptable amount of morbidity without a significant complication [15]. A different surgical approach was proposed in 2014. Perabo` et al. described a report of six patients who underwent concurrent mastectomy with RRSO being performed via a transmammary route with no significant complications. However, this is a not widespread surgical approach for RRSO with difficult to propose as a commonly procedure [22].

In literature, two interesting case-control studies were published with a control group who underwent mastectomies without RRSO. The two studies showed the safety of performing combined procedures with perforator-flap breast reconstruction [16,23] with no significant differences in anastomotic complications, fat necrosis, or seroma. Adding, Del Corral et al. performed a cost analysis demonstrating the costs reduction in combined surgery [23]. The largest study in literature concluded that combined procedures presented an acceptable morbidity [24]. So, compared to previous trials, our results confirmed that the combined surgery did not significantly increase the risk for postoperative breast complications and did not represent a specific risk for abdominal complications.

In terms of the secondary endpoint, our study was the first in the literature to assess patient satisfaction, postoperative pain, and recovery time. In our populations, 90% of patients were satisfied with the combined surgery, 95% agreed that the combined surgery did not increase postoperative pain, and 93% said they would have it done again.

The main strength of the present study is that this is one of the largest trials on this topic and it was the first trial that evaluated also patients’ satisfaction of the combined procedures. Other points of strength are certainly related to rigorous data collection methodology and strict inclusion criteria; all information were collected from our electronic hospital records, which are compiled by clinicians at each step of patient’s treatment. We included exclusively patients referred from diagnosis to treatment to our institute, excluding any sources of bias related to heterogeneous surgical choices and procedures. Despite these strengths, our results have some limitations deriving by the retrospective nature of the study and by the fact that our institution presented a long history of interdisciplinary combined surgery, therefore its applicability to other settings is unclear. Finally, another limitation is represented by the lack of a cost-efficacy analysis. However, concerning this last point it is clear that with the same complication rate and expenses related to their management, one hospitalization with two combined surgical procedures with a single anaesthesia versus two hospitalizations with two separate surgical procedures and anaesthesia, can represent a sure saving for the institution.

## 5. Conclusions

Breast surgery combined with RRSO for BRCA1–2 mutation carriers is a feasible and safe approach that reduces the number of hospitalizations and anesthetic administrations while allowing a single patient to recover postoperatively without increasing morbidity. Considering the high patient satisfaction, combined surgery is a viable option for patients with BRCA 1–2 mutation carriers. Certainly, prospective large studies with adequate long follow-up and evaluation of the protective effect against BC with RRSO are still mandatory.

## Figures and Tables

**Table 1:** Patients general features.

	BRCA 1 Carriers (*n* = 32)	BRCA 2 Carriers (*n* = 22)	Total (*n* = 54)
Mean Age at surgery	45 (35–72)	47 (42–63)	46 (35–72)
Patients without Breast Cancer	15 (27.7%)	11 (20.4%)	26 (48.1%)
Patients with current and/or recurrent Breast Cancer	17 (31.5%)	11 (20.4%)	28 (51.9%)
Menopausal Status			
Pre-menopausal	21 (38.9%)	9 (16.7%)	30 (55.6%)
Post-menopausal	11 (20.4%)	13 (24.1%)	24 (44.5%)
Familiarity			
Ovarian Cancer	18 (33.3%)	9 (16.7%)	27 (50.0%)
Breast Cancer	19 (35.2%)	18 (33.3%)	37 (68.5%)
Negative	3 (5.6%)	4 (7.4%)	7 (13.0%)

**Table 2:** Breast and gynecologic procedures performed.

	BRCA 1 Carriers (*n* = 32)	BRCA 2 Carriers (*n* = 22)	Total (*n* = 54)
Breast surgery: Bilateral Mastectomy	28 (51.8%)	19 (35.2%)	47 (87.0%)
RRBM	14 (25.9%)	8 (14.8%)	22 (40.7%)
Therapeutic	14 (25.9%)	11 (20.4%)	25 (46.3%)
Immediate reconstruction with implant	25 (46.3%)	16 (29.6%)	41 (75.9%)
With axillary staging	14 (25.9%)	7 (13.0%)	21 (38.9%)
Breast surgery: Unilateral Mastectomy	4 (7.4%)	3 (5.6%)	7 (13.0%)
Prophylactic	1 (1.8%)	3 (5.6%)	4 (7.4%)
Therapeutic	3 (5.6%)	0	3 (5.6%)
Immediate reconstruction with implant	3 (5.6%)	3 (5.6%)	6 (11.2%)
With axillary staging	2 (3.7%)	0	2 (3.7%)
Gynecologic surgery			
RRSO	28 (51.9%)	20 (37.0%)	48 (88.9%)
RRSO + LH	4 (7.4%)	2 (3.7%)	6 (11.2%)

Legend: RRBM: risk-reducing bilateral mastectomy; RRSO: Risk-reducing salpingo-oophorectomy; LH: laparoscopic hysterectomy.

**Table 3:** Surgery.

	Total (*n* = 54)
Median operative time mastectomy + RRSO, min	232 (150–283)
Median operative time RRSO, min	46 (32–60)
Median operative time mastectomy + RRSO + LTH, min	278 (200–345)
Median operative time RRSO + LH, min	105 (90–140)
Median hospital stays, days	3 (1–9)

Legend: RRSO: Risk-reducing salpingo-oophorectomy; LTH: laparoscopic total hysterectomy; LH: laparoscopic hysterectomy.

**Table 4:** Complications.

	Total Patients (*n* = 54)
Intraoperative complications	0
Total Postoperative complications <30 days	10 (18%)
Breast Postoperative complications <30 days	9 (16.6%)
Non-infectious reconstructive complication with removal expander/implant	1 (1.9%)
Infectious reconstructive complications with removal expander/implant	3 (5.6 %)
Infectious reconstructive complications without removal expander/implant	1 (1.9%)
Breast hematoma requiring evacuation	2 (3.7%)
Breast seroma aspiration	1 (1.9%)
Fever and Skin infection	1 (1.9%)
Gynecology Postoperative complications <30 days	1 (1.9%)
Trocar hematoma	1 (1.9%)
Total Postoperative complications >30 days	0

**Table 5:** Questionnaire.

Question 1	Very unsatisfied	Unsatisfied	Neutral		Satisfied	Very satisfied
Answers *n* (%)	42 (77%)	7 (13%)	2 (3%)		3 (7%)	0
Question 2	Yes			No		
Answers *n* (%)	3 (5%)			51 (95%)		
Question 3	Not very much	Not much	Neutral		Much	Very much
Answers *n* (%)	42 (77%)	7 (13%)	4 (8%)		0	1 (2%)
Question 4	Yes			No		
Answers *n* (%)	6 (12%)			48 (88%)		
Question 5	Yes			No		
Answers *n* (%)	50 (92%)			4 (8%)		

## Data Availability

Upon request.
